# The Orai1-AC8 Interplay: How Breast Cancer Cells Escape from Orai1 Channel Inactivation

**DOI:** 10.3390/cells10061308

**Published:** 2021-05-25

**Authors:** José Sánchez-Collado, José J. López, Juan A. Rosado

**Affiliations:** Cellular Physiology Research Group, Department of Physiology, Institute of Molecular Pathology Biomarkers, University of Extremadura, 10003 Caceres, Spain; josesc@unex.es

**Keywords:** Orai1, AC8, triple-negative breast cancer cells, inactivation, SOCE

## Abstract

The interplay between the Ca^2+^-sensitive adenylyl cyclase 8 (AC8) and Orai1 channels plays an important role both in the activation of the cAMP/PKA signaling and the modulation of Orai1-dependent Ca^2+^ signals. AC8 interacts with a N-terminal region that is exclusive to the Orai1 long variant, Orai1α. The interaction between both proteins allows the Ca^2+^ that enters the cell through Orai1α to activate the generation of cAMP by AC8. Subsequent PKA activation results in Orai1α inactivation by phosphorylation at serine-34, thus shaping Orai1-mediated cellular functions. In breast cancer cells, AC8 plays a relevant role supporting a variety of cancer hallmarks, including proliferation and migration. Breast cancer cells overexpress AC8, which shifts the AC8-Orai1 stoichiometry in favor of the former and leads to the impairment of PKA-dependent Orai1α inactivation. This mechanism contributes to the enhanced SOCE observed in triple-negative breast cancer cells. This review summarizes the functional interaction between AC8 and Orai1α in normal and breast cancer cells and its relevance for different cancer features.

## 1. Introduction

After the activation of cell membrane receptors, a cascade of intracellular signals ensures the connection between the stimulus and the cellular responses. Calcium and cAMP signals constitute the axis of two ubiquitous signaling mechanisms and control a myriad of cellular functions. Cytosolic cAMP concentration is regulated by the equilibrium between the activity of adenylyl cyclases (AC) and phosphodiesterases. However, Ca^2+^ is not generated or metabolized; the changes in cytosolic free-Ca^2+^ concentration ([Ca^2+^]_c_) are mediated by Ca^2+^ mobilization through Ca^2+^ channels and transporters. Resting cytosolic Ca^2+^ concentration is maintained at about 100 nM, while Ca^2+^ concentration in the endoplasmic reticulum (ER) is in the micromolar range, and that in the extracellular fluid ranges from 1.5 to 2.0 mM [1]. The resulting concentration gradient ensures sufficient Ca^2+^ release from the ER and Ca^2+^ influx from the extracellular medium for signal transduction when Ca^2+^ permeable channels open. Cell regulation of the magnitude and the spatiotemporal properties of the Ca^2+^ signals is vital for the versatility of this universal pathway [2]. Calcium signals’ shape and localization is managed by modulating the activity of Ca^2+^ channels, pumps and exchangers, which usually involves the concomitant activation of another signaling pathway, such as cAMP [3]. Apart from the reciprocal negative regulation between Ca^2+^ and cAMP, the interaction between these signaling factors originates cases of synergism where common effects are promoted [4].

## 2. Overview of Store-Operated Ca^2+^ Entry

Store Operated Ca^2+^ Entry (SOCE) is a ubiquitous signaling mechanism initiated after the depletion of ER Ca^2+^ stores. Under physiological conditions, SOCE is associated with the activation of membrane receptors, which causes the mobilization of ER Ca^2+^ reservoirs via IP_3_-receptor activation [5,6]. The first store-operated current identified, the Ca^2+^ release-activated current (*I*_CRAC_), is non-voltage-activated, inwardly rectifying, and highly selective for Ca^2+^ [5,6]. The key molecular components of SOCE are STIM and Orai proteins. STIM1 and its homolog STIM2 act as ER Ca^2+^ sensors and play an essential role in SOCE. The STIM proteins contain a cytosolic STIM-Orai-activating region (SOAR), which is the minimal sequence sufficient to activate Orai channels [7,8,9,10,11]. Upon store depletion, STIM proteins trigger Ca^2+^ influx from the extracellular medium by contacting and activating the store-operated Ca^2+^ channels [12]. The Ca^2+^-released activated Ca^2+^ (CRAC) channels are comprised of Orai1 [13,14,15,16], which is highly selective to Ca^2+^. Initially, the involvement of the Orai1 homologs, Orai2 and Orai3, in CRAC channels was unclear; however, recently, native CRAC channels have been described as hexameric structures formed by the association between different Orai family members, leading to channels that fine-tune the extent of SOCE to match the strength of the stimulation with different concentrations of physiological agonists [17,18,19]. In addition to *I*_CRAC_, another store-operated current, less selective for Ca^2+^, has been described, *I*_SOC_, which involves the participation of Orai1, the canonical transient receptor potential (TRP) channel TRPC1 and STIM1 [20,21,22]. Whether TRPC1 is able to operate as a store-operated channel in the absence of Orai1 still remains controversial. While there is some overlap between the Ca^2+^ currents generated by Orai1 and TRPC1, they have been reported to regulate different cell functions [23] and, in this review, we will focus on the regulation of Orai1 channels.

In its resting state, STIM1 exists as a dimer, in a quiescent configuration, with the SOAR domain occluded in the folded C-terminal region. Calcium store depletion leads to dissociation of Ca^2+^ from the STIM1 EF-hand domain, leading to STIM1 multimerization and translocation to puncta within the ER membrane located in ER-plasma membrane (PM) junctions [24]. Subsequently, STIM1 undergoes a conformational change, leading to association of the EF-hand/SAM domains in the intraluminal N-terminal region of the STIM1 dimer [25]. The conformational change is transferred through the transmembrane domains and unbends the cytosolic region, leading to exposure of the SOAR to bind to and activate the Orai1 channel. The molecular model for the interaction with and activation of Orai1 by STIM1 predicts the unimolecular coupling between STIM1 and Orai1 and suggests that the available STIM1 subunit of the dimer might undergo inter-hexameric Orai1 channel crosslinking [26]. The Orai1 leucine-273 located in the Orai1 C-terminal STIM1-binding domain has been reported to play an essential role in Orai1 gating by STIM1 [27], propagating the STIM1-binding signal through the Orai1 transmembrane domains to the pore-forming helices to induce channel gating [28].

Native CRAC channels have been reported to be formed by the heterogeneous association of Orai proteins, which confer CRAC channel differences in their biophysical properties. For instance, Orai3 has been reported to modulate inhibition of *I*_CRAC_ by reactive oxygen species [29,30,31]. In immune cell types, Orai2, together with Orai1, is a primary component of the CRAC channels and plays a relevant functional role [32,33]. Furthermore, Orai2 and, especially, Orai3 exhibit more prominent fast Ca^2+^-dependent inactivation than Orai1 [34]. Based on the stronger Ca^2+^-dependent inactivation, Orai2 and Orai3 negatively regulate CRAC currents and might modulate native Ca^2+^ signals and the subsequent cellular responses, emphasizing Orai proteins’ expression and stoichiometry as crucial factors to understand the role of SOCE in a given cell type [18,19].

## 3. Orai1 Variants

**Figure 1:**
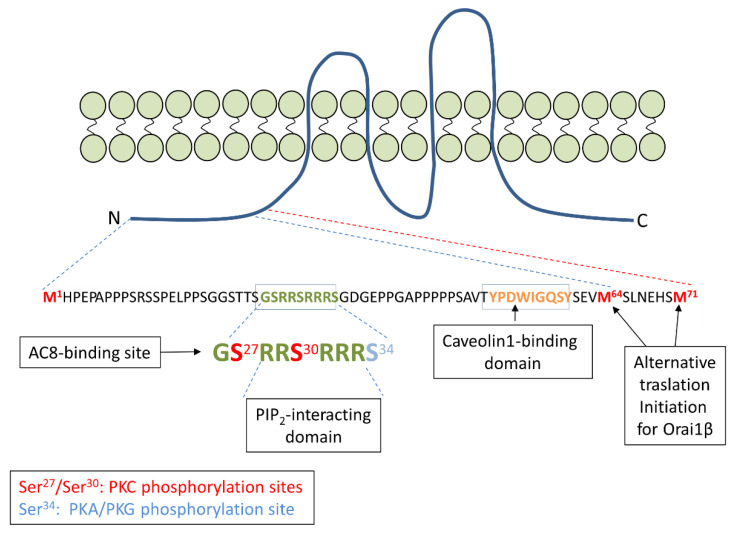
Amino acid sequence of the N-terminal region unique to Orai1α. Within the 63 or 70 residues unique for the N-terminal region of Orai1α there are several functional domains, including one of the caveolin1-binding domains and the AC8-binding site, which overlap with the PIP_2_-interacting region and the PKC and PKA/PKG phosphorylation sites (serines 27, 30 and 34, respectively).

In 2012, Fukushima et al. [35] reported the existence of two Orai1 variants at the protein level in mammalian cells (reviewed in [36]). The longer variant, Orai1α, is the full-length Orai1 with 301 amino acids, while the short variant, termed Orai1β (231–238 amino acids), arises by a process of alternative translation initiation from a methionine at position 64 (probably also 71) in the Orai1α variant. Therefore, Orai1β lacks the N-terminal amino acids 1 to 63 or 70 that exist in Orai1α, which exhibits different motifs with potential functional relevance (Figure 1). Specifically, the sequence between amino acids 26–34 in Orai1α is involved in its interaction with the Ca^2+^-regulated adenylyl cyclase 8 (AC8) [37]. In addition, the serine residues at positions 27 and 30 are PKC phosphorylation sites, and phosphorylation of Orai1 at serines 27 and 30 has been associated with channel inactivation [38]. Serine-34 has been reported as a PKG [39], as well as PKA [40], phosphorylation site; phosphorylation of this residue is also involved in Orai1α inactivation. There is a predicted PIP_2_-binding domain in the polyarginine sequence between amino acids 28–33 [38]. Finally, the sequence between amino acids 52–60 forms a caveolin-binding domain [41]. While Orai1α and Orai1β show similar subcellular localization and both isoforms are able to support both *I*_CRAC_ and *I*_SOC_ currents [20,35], they exhibit some functional differences. Among them, Orai1α, but not Orai1β, is able to support the *I*_ARC_ current [20], a non-capacitive Ca^2+^ current through the arachidonate acid-regulated Ca^2+^ (ARC) channel and involving the participation of Orai1, Orai3 and the minor pool of plasma membrane resident STIM1 [42,43,44]. Furthermore, Orai1α exhibits a more pronounced fast Ca^2+^-dependent inactivation [20], and FRAP recovery experiments have revealed that Orai1α shows slower plasma membrane mobility than Orai1β [35].

Orai1α and Orai1β have been reported to activate NFAT1 nuclear translocation upon cell stimulation with thapsigargin [40]. However, a recent study has revealed that Orai1α interacts with the scaffold protein A-kinase anchoring protein (AKAP)-79, while Orai1β shows a weak association with this protein [45]. AKAP-79 has been reported to allow colocalization between NFAT1 and the Ca^2+^-dependent phosphatase calcineurin, which facilitates NFAT1 dephosphorylation [46]. The findings by Kar and coworkers challenge the participation of Orai1β in NFAT1 nuclear translocation or suggest an alternative pathway for Orai1β-mediated NFAT1 activation [45]. The presence of two Orai1 variants with slightly different biophysical properties, together with the different expression of Orai2, Orai3 and STIM isoforms and variants, unveils the heterogeneity of agonist-stimulated Ca^2+^ signals.

## 4. Orai1-Interacting Proteins: Adenylyl Cyclase 8

**Figure 2:**
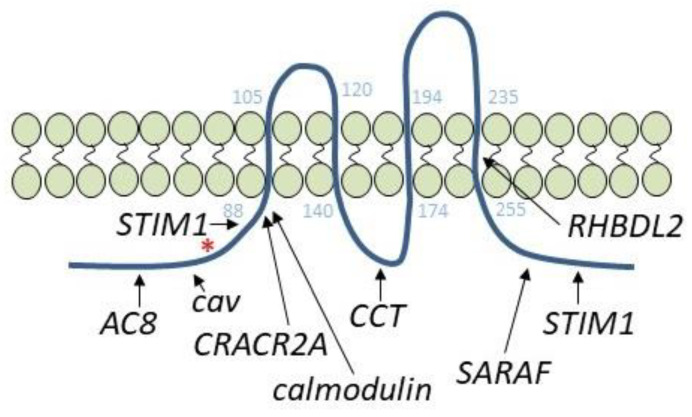
Cartoon depicting the most relevant Orai1-interacting proteins: AC8, adenylyl cyclase isoform 8; cav, caveolin-1; CRACR2A, CRAC regulator 2A; CCT, chaperonin-containing TCP-1; SARAF, SOCE-associated regulatory factor; RHBDL2, rhomboid-like 2. The residues that delimit the transmembrane domains are represented in light blue. The asterisk indicates the start of Orai1β.

Besides STIM1, different proteins have been reported to interact with and regulate Orai1 activation and function (Figure 2). Among them, CRACR2A (also known as CRAC regulator 2A, EFCAB4B or FLJ33805) is a cytosolic protein that contains two EF-hand domains in the N-terminal region and stabilizes the STIM1-Orai1 interaction. CRACR2A interacts with the N-terminal region of Orai1, involving lysines at positions 85 and 87, and this interaction plays a relevant role in Orai1 clustering and activity [47]. Nevertheless, the more recent publication of the crystal structure of the full *Drosophila melanogaster* Orai1, showing that the pore of the hexameric channel, which extends > 20 Å into the cytosol, includes lysines 85 and 87 [17], challenges the possibility that CRACR2A directly interacts with those residues according to the structure of the *Drosophila* Orai1. The chaperonin-containing TCP-1 (CCT) is a molecular chaperone that interacts with the sequence between amino acids 157–167, located in the Orai1 intracellular loop; this increases PM residence of Orai1, leading to faster STIM1-Orai1 puncta formation [48]. The STIM1-modulator SARAF (SOCE-associated regulatory factor) has been demonstrated to activate Orai1 by its interaction with the C-terminus of Orai1 [49]. SARAF is a 339-amino-acid-long protein that has been identified as a STIM1-interacting protein that modulates SOCE and prevents Ca^2+^ overload. SARAF interacts with the C-terminal inhibitory domain of STIM1, preventing spontaneous activation of STIM1 and modulating basal ER and cytosolic Ca^2+^ concentrations [50]. The dynamic interaction between SARAF and STIM1 has been shown to be modulated by the Ca^2+^-binding protein EFHB (EF-hand domain family member B [51]); Caveolin-1 has also found to interact with and regulate Orai1 function. A number of studies have provided evidence for a positive role of caveolin-1 in the activation of SOCE [52,53]. Two caveolin-binding sites have been reported in Orai1 located between amino acids 52 and 60, in the N-terminal region, and at residues 250 to 253, located in the fourth transmembrane domain [41,54]. Orai1 internalization has been found to involve the caveolin-binding site located between residues 52 and 60, that exists in Orai1α exclusively [55], and a more recent study has revealed that the cytosolic C-terminal sequence between amino acids 260–275 is also essential for Orai1 internalization in *Xenopus* oocytes during meiosis [56]. A different mechanism for Orai1 endocytosis has been described in renal proximal tubular epithelial cells, where Orai1 colocalizes with clathrin instead of caveolin after interaction with amnionless, a protein associated with receptor endocytosis, leading to clathrin-mediated endocytosis of Orai1/STIM1 complexes and albumin upon Ca^2+^ store depletion [57]. Other proteins that regulate the Orai1 channel function include ubiquilin, which downregulates SOCE by promoting the ubiquitination and lysosomal degradation of Orai1 [58], and the recently reported RHBDL2 protein, a rhomboid intramembrane protease that interacts with the fourth transmembrane domain of Orai1 and degrades inappropriately activated CRAC channels in non-stimulated cells [59]. In contrast to the molecular mechanisms leading to a reduction of Orai1 plasma membrane expression, different pathways have been reported to enhance Orai1 surface exposure. In this context, secretory pathway Ca^2+^-ATPase (SPCA2) has been shown to interact with Orai1 and promote its translocation to the plasma membrane, leading to constitutive channel activation [60,61,62].

Recently, Zhang et al. have described the role of AC8 in Orai1 Ca^2+^-dependent inactivation [40]. As mentioned above, the N-terminal domain of AC8 interacts with the sequence GSRRSRRRS (amino acids 26–34) located in the N-terminal region of Orai1 [37]. The AC8-binding region is absent in the short Orai1 variant, Orai1β, since unlike the long Orai1 variant, Orai1α, it lacks the N-terminal 63 amino acids. As a consequence, AC8 does not interact with Orai1β [63]. Calcium entry through Orai1 triggers AC8 activity, which, in turn, increases cytosolic cAMP concentration in the channel microenvironment [64], thus providing a point of convergence for Ca^2+^ and cAMP signals.

AC8 is among the Ca^2+^-sensitive adenylyl cyclases. Despite the lack of EF-hand motifs in AC8, it contains two calmodulin (CaM)-binding domains, located in the N-terminal domain and the C2b region [65]. In resting cells, AC8 is in a quiescent/autoinhibited state supported by steric hindrance caused by the C-terminal domain that prevents ATP interaction with the ATP-binding site, and upon cell stimulation the enzyme is activated by a Ca^2+^ and CaM-dependent conformational change [66]. The first evidence supporting the activation of AC8 by Ca^2+^ entry was provided by Fagan et al. [67], reporting that AC8 was robustly stimulated by SOCE but not by Ca^2+^ released from the intracellular stores or by Ca^2+^ influx elicited by Ca^2+^ ionophores. Subsequent studies demonstrated that the activation of AC8 by SOCE occurred both in electrically excitable and non-excitable cells, the former also showing activation of AC8 by Ca^2+^ influx elicited by the opening of L-type (Ca_v_1.2) Ca^2+^ channels [68,69]. Nevertheless, in non-excitable cells AC8 activation has been reported to occur exclusively upon activation of SOCE, since non-capacitative Ca^2+^ influx through the ARC channels or diacylglycerol-activated TRPC channels failed to activate AC8 [70,71].

Once activated, AC8 induces local increases in cAMP, which, in turn, activate PKA, leading to the phosphorylation of Orai1 at serine-34, a mechanism specific to Orai1α. Co-localization of PKA with Orai1 is mediated by AKAP-79 [40]. Phosphorylation of Orai1α at serine-34 leads to channel inactivation and shapes the Orai1-mediated Ca^2+^ signals and function. Specifically, while both Orai1 variants can activate nuclear translocation and transcriptional activity of NFAT4, an NFAT isoform sensitive to small changes in [Ca^2+^]_c_, upon stimulation with physiological agonist concentrations Orai1β-mediated Ca^2+^ signals supported faster and more robust NFAT4 translocation than those mediated by Orai1α [40]. These findings suggest that the relative expression of Orai1α and Orai1β, and the abundance of AC8, in a given cell type might significantly remodel Ca^2+^ signals and functions evoked by physiological concentrations of agonists.

## 5. Orai1-Adenylyl Cyclase 8 in Cancer Cells

Cancer cells are characterized by the disturbance of the fine balance of the cell cycle, shifting this balance towards excessive proliferation while attenuating pathways leading to cell death. In cancer cells, crucial processes leading to proliferation—but also migration, invasion and drug or apoptosis resistance—are mediated by Ca^2+^ and cAMP [63,72]. Some cancers, in order to orchestrate the oncogenic machinery, tune the signaling pathways by modifying the expression of key regulators or by favoring specific post-translational protein modifications [73], thus remodeling the signal transduction mechanisms. Breast cancer is a heterogeneous disease that is commonly classified by its histological and molecular features and gene expression profile into luminal, HER2 and triple-negative types [74]. The latter has been stratified into six subtypes known as basal-like 1 (BL1), basal-like 2 (BL2), mesenchymal (M), mesenchymal stem-like (MSL), immunomodulatory (IM), and luminal androgen receptor (LAR) [75,76].

In recent decades, numerous studies have described the link between Ca^2+^ signals and the hallmarks of breast cancer, revealing changes in protein expression and activity with a clear heterogeneity among cancer subtypes. In the context of SOCE, Ca^2+^ entry through CRAC channels promotes epithelial to mesenchymal transition, cell proliferation, angiogenesis, metastasis and resistance to chemotherapy in breast cancer cells [77]. Supporting this crucial role, Orai1 overexpression has been described in a variety of breast cancer subtypes, showing a dominant function in triple-negative breast cancer (TNBC) cells [78,79]. Conversely, STIM1 expression level shows a wide heterogeneity which embraces differences among cell lines from the same cancer subtype. Interestingly, in breast cancer cells of the luminal subtype (estrogen receptor positive (ER+) breast cancer cells), SOCE is strongly dependent on Orai3 channels, whose expression depends on estrogen receptor-α in these cells [80], with Orai1 playing a minor role [78]. These observations illustrate the heterogeneity of Ca^2+^ handling in cells from different breast cancer subtypes.

**Figure 3:**
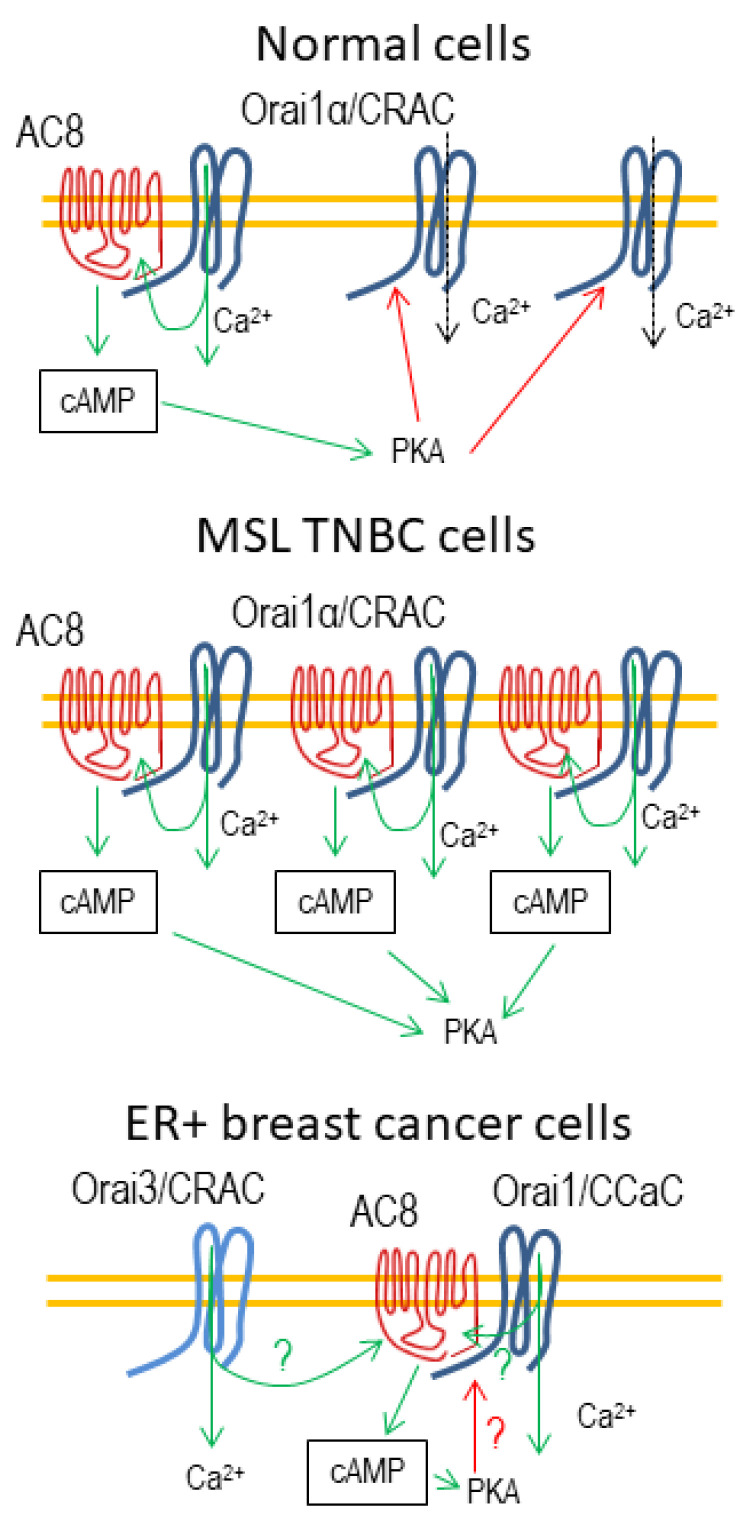
Cartoon depicting the AC8-Orai interplay in normal and breast cancer cells. Only one Orai subunit of the CRAC channel hexameric structure is depicted for clarity. In normal cells, Orai1 plays a predominant role in CRAC channels. Ca^2+^ influx through Orai1α leads to the activation of AC8, which in turn results in the activation of cAMP-dependent protein kinase (PKA) and inactivation of the AC8-free Orai1α subunits by phosphorylation at serine-34, thus moderating SOCE. In mesenchymal stem-like (MSL) TNBC cells Orai1 is the major component of CRAC channels. In these cells, AC8 overexpression decreases the number of AC8-free Orai1α subunits susceptible to phosphorylation and inactivation, leading to enhanced SOCE. In ER+ breast cancer cells, SOCE is mediated by Orai3, which is unable to bind AC8, and Orai1 participates in SPCA-dependent constitutive Ca^2+^ entry (CCaC). AC8 activation is activated by still unknown mechanisms that might involve Ca^2+^ influx via Orai1 or Orai3 in the close vicinity of Orai1 channels. The possible inactivation of Orai1-forming CCaCs by PKA remains unknown.

Based on the previous studies by Willoughby [37] and Zhang [40] in non-tumoral cells, in 2019 we provided evidence for the remodeling of the Orai1-AC8 interplay in different subtypes of TNBC cells and its role in the promotion of breast cancer hallmarks [63]. As mentioned earlier, AC8-Orai1 coupling allows two sequential functions: (1) the activation of AC8 upon Ca^2+^ influx through the channel, leading to the activation of the cAMP signaling pathway, and (2) the subsequent inactivation of Orai1 by PKA phosphorylation at serine-34. Concerning the regulation of Orai1 by the AC8/PKA pathway, two considerations should be taken into account. First of all, Willoughby and coworkers observed that the interaction of AC8 with Orai1 occurs at the AC8-binding site in the unique N-terminal region of Orai1α [37], and that the phosphorylation sites of PKC, PKA and PKG at serines 27, 30 and 34, with relevant roles for channel inactivation, are also found at the exclusive N-terminus of Orai1α [38,39,40]. These findings reveal that AC8 does not interact with the short Orai1 variant, Orai1β, as demonstrated experimentally [63], and that Orai1β is not susceptible to inactivation by phosphorylation of the mentioned serine residues. The AC8-mediated Orai1 inactivation is expected to solely affect the Orai1α-forming CRAC channels; the existence of Orai1α:Orai1β heteromeric channels remains to be elucidated. Second, the AC8-binding site overlaps with serines-27, 30 and 34 (see Figure 1), and, therefore, AC8 binding might interfere with phosphorylation at the mentioned serines. In triple-negative MDA-MB-231 breast cancer cells of the MSL subtype [75], both Orai1 variants as well as AC8 show an enhanced expression at the protein level, but these cells predominantly express AC8, which shifts the Orai1α/AC8 stoichiometry in favor of AC8 [63]. In MDA-MB-231 cells, determination of the serine phosphorylation status of Orai1 at native conditions revealed that the AC8-bound Orai1 subunits (Orai1 that co-immunoprecipitates with AC8) are not phosphorylated at serine residues, while the AC8-free Orai1 subunits show a significant serine phosphorylation, thus providing evidence for the impairment of Orai1 serine phosphorylation upon AC8 binding [63]. These findings were confirmed in cells transfected with siRNA for AC8 gene silencing or AC8 overexpression plasmid, where AC8 knockdown enhances Orai1 serine phosphorylation while AC8 overexpression abolishes this process. In a model where interaction with AC8 prevents PKA-dependent phosphorylation and inactivation of a given Orai1 subunit, one would postulate that overexpression of AC8, as observed in MDA-MB-231 cells, might decrease the number of channels not associated with this cyclase, thus impairing phosphorylation-dependent CDI and subsequently enhancing Ca^2+^ influx (Figure 3). Our results indicated that AC8 knockdown in MDA-MB-231 cells significantly attenuated TG-induced SOCE as well as Ca^2+^ influx evoked by co-expression of Orai1α and the Orai1-activating small fragment (OASF) region of STIM1 [9], which activates CRAC channels independently of Ca^2+^ store depletion, and, conversely, AC8 overexpression slightly but significantly enhances SOCE and Ca^2+^ influx mediated by co-expression of OASF and Orai1α in these cells [63]. These findings indicate that, while in normal cells the interaction of AC8 with Orai1α leads to net channel inactivation [40], in TNBC cells of the MSL subtype, overexpression of AC8 interferes with Orai1α phosphorylation and inactivation, resulting in enhanced SOCE.

Remodeling of the AC8 expression, and thus AC8-Orai1α stoichiometry, was proved to be important for the support of certain SOCE-dependent cancer hallmarks. AC8 expression attenuation reduced proliferation in the MSL-TNBC cell lines MDA-MB-231 and Hs578T, and inhibited MDA-MB-231 cell migration by inhibition of tyrosine phosphorylation of the focal adhesion kinase (FAK) [63]. Although the effect observed after AC8 silencing might be attributed to the regulation of SOCE, a functional role for the cAMP pathway in these processes cannot be ruled out. There is no information about the functional interaction between Orai1α and AC8 in other subtypes of TNBC cells, and the different gene expression profile between subtypes prevents the generalization of the findings observed in MSL cells, but given the potential interest of these findings to identify new pharmacological targets for the treatment of TNBC, the analysis of the interaction between Orai1 and AC8 in other TNBC subtypes deserves further study.

AC8 overexpression also occurs in estrogen receptor positive (ER+), luminal A, breast cancer MCF7 cells, together with a high expression of Orai1α and Orai1β [63]. Nevertheless, in these cells, Orai3 is sufficient to conduct SOCE, as Orai3 knockdown impairs TG-induced Ca^2+^ influx, while knockdown of either Orai1 or Orai2 was without effect on SOCE in MCF7 cells [78]. It is unclear whether Orai1 or Orai2 are constituents of the CRAC channels in MCF7, with Orai3 as the predominant subunit, but if this proves to be the case, their role might be redundant. However, a role for Orai1 has been reported in constitutive Ca^2+^ entry in these cells stimulated by SPCA [60]. Whether SPCA can indistinctly interact with Orai1α and Orai1β, as well as the possible role of AC8 in the regulation of constitutive Ca^2+^ entry mediated by Orai1, remains to be elucidated. As the AC8-binding site is exclusive for Orai1α and, therefore, AC8 is unable to associate to Orai2 and Orai3 [37], it is quite unlikely that AC8 regulates SOCE in MCF7 cells and, therefore, the mechanism described above is cancer subtype-specific. Nevertheless, AC8 plays a functional role in MCF7 cells, as we have found that AC8 knockdown leads to attenuation of MCF7 cell migration to a similar extent as pharmacological inhibition of PKA both in resting conditions and upon stimulation with carbachol, a Ca^2+^-mobilizing agonist [63]. These findings indicate that AC8 can still be activated in MCF7, probably by interaction with Orai1-forming, constitutively open Ca^2+^ channels or by proximity to Orai3-forming CRAC channels (Figure 3). Activated AC8 might result in the generation of cAMP and subsequent activation of PKA and other downstream effectors, including the transcription factors CREB and CREM or the exchange protein directly activated by cAMP (Epac) [81]. We have found that the increase in cytosolic cAMP using the cell-permeant 8-Br-cAMP enhances MCF7 cell migration, and, conversely, inactivation of PKA by KT-5720 attenuates cell migration [63]. Furthermore, pharmacological inhibition of Epac has been reported to disrupt its association with the microtubule cytoskeleton, to induce delocalization of AKAP9 from the centrosome, and to result in attenuation of MCF7 cell migration, leading to cell death [82]. Altogether, these findings indicate that activation of the cAMP pathway in estrogen-receptor-positive MCF7 breast cancer cells plays a relevant role in cell migration and tumorigenesis.

## 6. Conclusions

In summary, the AC8-Orai1α interplay is a relevant mechanism for CRAC channel inactivation and for shaping the Ca^2+^ signals in response to physiological agonists. In TNBC cells of the MSL subtype, the expression of AC8 and Orai1α is altered to shift the AC8-Orai1α stoichiometry in favor of the former, thus reducing the number of non-AC8-bound Orai1α subunits susceptible to inactivation and, as a result, enhancing SOCE and supporting a variety of cancer hallmarks. While a role for AC8 in the regulation of SOCE in estrogen receptor positive breast cancer cells is unlikely, AC8 is overexpressed in these cells, and its activation by Ca^2+^-dependent mechanisms plays a relevant role in the development of cancer features. Unveiling the mechanisms underlying breast cancer cell biology will shed a light on the identification of novel pharmacological opportunities for cancer therapy, inspiring the development of specific anti-tumoral strategies.

## Data Availability

The study does not report any new data.
